# The experience of tobacco withdrawal symptoms among current smokers and ex-smokers in the general population: Findings from nationwide China Health Literacy Survey during 2018-19

**DOI:** 10.3389/fpsyt.2022.1023756

**Published:** 2023-01-13

**Authors:** Zi-yang Cui, Ying-hua Li, Zhao Liu, Li Li, Xue-qiong Nie, Xin-mei Zhou, An-qi Cheng, Jin-xuan Li, Rui Qin, Xiao-wen Wei, Liang Zhao, Daniella Ladmore, Francesca Pesola, Kian Fan Chung, Zheng-ming Chen, Peter Hajek, Dan Xiao, Chen Wang

**Affiliations:** ^1^Department of Tobacco Control and Prevention of Respiratory Diseases, Center of Respiratory Medicine, China-Japan Friendship Hospital, Beijing, China; ^2^WHO Collaborating Center for Tobacco Cessation and Respiratory Diseases Prevention, Beijing, China; ^3^National Clinical Research Center for Respiratory Diseases, Beijing, China; ^4^Institute of Respiratory Medicine, Chinese Academy of Medical Sciences, Beijing, China; ^5^National Center for Respiratory Medicine, Beijing, China; ^6^Graduate School of Peking Union Medical College, Beijing, China; ^7^China Health Education Center, Beijing, China; ^8^China-Japan Friendship School of Clinical Medicine, Capital Medical University, Beijing, China; ^9^Wolfson Institute of Population Health, Queen Mary University of London, London, United Kingdom; ^10^National Heart and Lung Institute, Imperial College London and Royal Brompton and Harefield Hospitals, London, United Kingdom; ^11^Nuffield Department of Population Health, University of Oxford, Oxford, United Kingdom; ^12^Peking Union Medical College, Chinese Academy of Medical Sciences, Beijing, China

**Keywords:** tobacco withdrawal symptom, smoking, smoker, risk factors, China Health Literacy Survey (CHLS)

## Abstract

**Objective:**

To clarify the extent to which smokers in the general population experience tobacco withdrawal symptoms and whether such experience differs in those who continue to smoke and those who stopped smoking.

**Methods:**

We included relevant questions in the nationally-representative China Health Literacy Survey (CHLS) conducted in 2018–2019. Among 87,028 participants, there were 22,115 ever-smokers aged 20–69 years who provided information on their smoking history and their experience of tobacco withdrawal symptoms. Multivariate logistic regressions were conducted to explore the association between withdrawal symptoms and other variables.

**Results:**

Among ever-smokers, there were 19,643 (88.8%) current smokers and 2,472 (11.2%) ex-smokers. Among current smokers, 61.3% reported having tried to quit smoking in the past. Overall, 61.1% of current smokers reported experiencing withdrawal symptoms: 69.9% of those who tried to quit smoking in the past and 47.5% of those who did not. A lower proportion of ex-smokers experienced withdrawal symptoms (46.3%) and the difference remained significant after controlling for demographic characteristics (OR = 1.76, 95% CI 1.62–1.93, *P* < 0.001). The most commonly reported withdrawal symptoms in both current smokers and ex-smokers were craving, restlessness and anxiety. In the multivariable-adjusted analyses, those who experienced withdrawal symptoms when they tried to quit smoking (OR: 2.05, 95% CI: 1.86–2.27) were less likely to successfully quit.

**Conclusions:**

The clinical picture of the tobacco withdrawal syndrome is the same in current smokers and in ex-smokers, but ex-smokers are less likely to have experienced it. The experience of discomfort when unable to smoke is common and seems likely to be a major factor contributing to maintaining smoking behavior not just among individuals seeking help with quitting smoking, but among smokers generally.

## Introduction

Stopping smoking is often accompanied by withdrawal discomfort, comprising of an array of tobacco withdrawal symptoms (1). In dependent smokers, tobacco abstinence is typically accompanied by urges to smoke, restlessness, hunger, irritability, and other adverse mood changes (2, 3). Other less frequent but occasionally severe tobacco withdrawal symptoms include insomnia (4), mouth ulcers (5), and constipation (6). The phenomenology of subjective tobacco withdrawal has been documented primarily in studies of smokers seeking help with quitting smoking, i.e., those at the extreme end of the tobacco dependence continuum. Although it is generally assumed that most smokers attempting to quit experience some withdrawal symptoms, little objective data exist on this issue. Another open question is whether within the general population, as opposed to clinical samples, successful quitters differ in their withdrawal experience from smokers who continue to smoke.

Available data on these issues provide only tentative and inconsistent answers. Among a random sample of 239 US smokers who tried to quit or cut down, withdrawal symptoms were more common and more severe in participants with a history of depression and anxiety. Most symptoms were reported by over half of the responders. Symptom severity was unrelated to continuing smoking (7). In another small study based on German population, 144 participants who did not succeed in quitting were compared with 84 who managed to stop smoking. Proportions of responders who reported experiencing 1, 2, 3, and 4 or more withdrawal symptoms were similar in the two groups (8). In a sample of US high school students, a higher proportion of current smokers than successful quitters reported experiencing withdrawal symptoms at their most recent quitting attempt (9).

To clarify the extent to which smokers in the general population experience tobacco withdrawal symptoms; and whether such experience differs in those who continue to smoke and those who stop smoking, we included questions on tobacco withdrawal symptoms in a large Chinese representative population-based survey, the China Health Literacy Survey (CHLS).

## Materials and methods

### Study design and participants

The CHLS is a nationally representative annual household survey, which started in 2012 and collects data from non-institutionalized men and women aged 15–69 years. Details of CHLS have been reported elsewhere (10).

The sampling procedure of 2018 CHLS, shown in Supplementary Figure 1, ensures a nationally-representative sample. All 31 provinces of China were stratified into urban and rural areas. The population proportionate sampling method (PPS) is used to randomly select 336 districts, with their population size estimated from China’s Sixth National Population Census (11). Next, the PPS method was used to randomly select 3 towns in each district, resulting in the inclusion of 1,008 towns and 2 villages in each district, resulting in the inclusion of 2,016 villages. A total of 55 households were randomly selected in each town and village, and the investigators collected the information from at least 40 residents aged 15–69 years. The final sampling was stratified based on the 2010 China Census data (11). Further details of study protocol are available in Appendix.

Between December 2018 and December 2019, 87,028 participants provided survey data, including 22,115 ever-smokers aged 20–69 years (see Supplementary Figure 2). We excluded individuals aged under 20 years as the younger sample did not meet representativeness criteria (*n* = 1,680) (Supplementary Figure 2).

### Procedures and measures

Trained health workers administered a standardized questionnaire at local community health centers. The questionnaires recorded sociodemographic characteristics, medical history, and lifestyle factors listed in Table 1.

**TABLE 1:** Demographic characteristics of study population.

Variables	Current smokers (*n* = 19,643)	Ex-smokers (*n* = 2,472)	*p*
Men *N* (%)	18,643 (94.90)	2,321 (93.90)	0.033
Age (year groups) *N* (%)			<0.001
20–29	1,375 (7.00)	102 (4.13)	
30–39	2,711 (13.80)	250 (10.11)	
40–49	4,894 (24.91)	449 (18.16)	
50–59	5,736 (29.20)	679 (27.47)	
60–69	4,927 (25.08)	992 (40.13)	
Median (IQR)	51.00 (18.00)	55.00 (17.00)	<0.001
Ethnicity *N* (%)			0.004
Han	17,682 (90.02)	2,271 (91.87)	
Others	1,961 (9.98)	201 (8.13)	
Residence *N* (%)			0.002
Urban	8,405 (42.79)	1,139 (46.08)	
Rural	11,238 (57.21)	1,333 (53.92)	
Marital status *N* (%)			<0.001
Single	1,449 (7.38)	109 (4.41)	
Married	16,671 (84.87)	2,175 (87.99)	
Separated/Divorced/Widowed	1,523 (7.75)	188 (7.61)	
Education *N* (%)			0.020
Primary school	6,669 (33.95)	801 (32.40)	
Middle or high school	10,850 (55.24)	1,360 (55.02)	
College and higher education	2,124 (10.81)	311 (12.58)	
Geographical region *N* (%)			<0.001
East	7,197 (36.64)	1,093 (44.22)	
Central	5,292 (26.94)	688 (27.83)	
West	7,154 (36.42)	691 (27.95)	
Annual household income (RMB) *N* (%)			<0.001
<20,000	6,143 (31.27)	707 (28.6)	
20,000–49,999	6,677 (33.99)	755 (30.54)	
≥50,000	6,823 (34.74)	1,010 (40.86)	
Median (IQR)	30,000 (46,000)	30,000 (45,000)	<0.001
Self-reported overall health status *N* (%)			<0.001
Good	11,310 (57.58)	1,225 (49.56)	
Average	6,968 (35.47)	960 (38.83)	
Poor	1,365 (6.95)	287 (11.61)	

Data are shown as number (%), median (IQR) or mean (SE).

The questions on smoking were taken from The Global Adult Tobacco Survey (GATS) (12, 13) and China Adult Tobacco Survey (14). Participants were classified as current smokers or ex-smokers based on the question: do you smoke cigarette? (A) I am currently smoking; (B) I used to smoke, but have given up; and (C) I have never smoked. Ever smokers were asked about number of cigarettes smoked per day, the duration of smoking, and the age of starting smoking. Fagerström Test of Nicotine Dependence (FTND) (15) was used to classify tobacco dependence as mild (0–3 points), moderate (4–6 points), or high (≥7 points).

Tobacco withdrawal symptoms were defined as adverse symptoms that smokers experience after stopping smoking. The occurrence of withdrawal symptoms was assessed by asking participants to indicate which symptoms, if any, from a list of symptoms they experienced when unable to smoke (16). The list was derived from existing literature (1, 6, 16–21) and included urge-to-smoke, restlessness, anxiety, difficulty concentrating, irritability/frustration/anger, dysphoric mood, insomnia, tiredness, weight gain, mouth ulcers, constipation, and others. The verbatim phrasing of the question was: Have you ever experienced these symptoms when you were unable to smoke?

### Statistical analysis

We summarized demographic characteristics using mean (SD) for continuous variables that are approximately symmetric, or median and interquartile range (IQR) if they are skewed, and used frequencies for categorical data. The characteristics of current smokers and ex-smokers were compared using independent-sample *t*-test or Mann–Whitney test for continuous variables and by the χ^2^ test for categorical variables. We used the design-based logistic regression (which includes sampling weights) to estimate the association between withdrawal symptoms (i.e., present vs. absent) and each potential predictor for current and ex-smokers, respectively. When exploring associations between withdrawal symptoms and predictors, in the first step, each predictor was assessed separately and those associated with withdrawal (*p* < 0.1) were then included in the final multivariate model. In the multivariable logistic regression for current smoker, withdrawal status was regressed onto age, sex, marital status, self-reported overall health status, smoking pack-years, age at starting smoking, FTND, failed attempts to quit or cut down. In the multivariable logistic regression for ex-smokers, withdrawal status was regressed onto age, sex, self-reported overall health status, smoking pack-years, age at starting smoking, FTND, failed attempts to quit or cut down. We report adjusted odd ratios, 95% confidence intervals (CI) and *p*-values from the Wald test for each predictor included in the final multivariate model. In addition, we used the method-based logistic regression as a sensitivity analysis. As some comparisons included multiple testing, the threshold for significance was set at a *p*-value of less than 0.01 (Bonferroni correction).

The analyses were conducted in SAS 9.4 (SAS Institute Inc., Cary, NC, USA). All reported *p*-values are two-sided.

## Results

Only 2.4% of participants provided responses that included missing data and their questionnaire were not included (Supplementary Figure 2).

### Sample characteristics

Of the 22,115 ever-smokers, 20,964 (94.8%) were men. Overall, 88.8% of ever-smokers were current smokers and 11.2% were ex-smokers. Demographic characteristics of current smokers and ex-smokers are shown in Table 1. Compared with current smokers, ex-smokers were older (*P* < 0.001), with higher education (*P* = 0.020), and poorer health (*P* < 0.001).

Supplementary Table 1 shows smoking characteristics of current smokers and recalled details of previous smoking in ex-smokers. The two groups started to smoke at similar age (when 20 years old), but during their smoking period, ex-smokers smoked fewer cigarettes per day (13 vs. 15) and showed fewer signs of tobacco dependence (2.94 vs. 3.46).

### Withdrawal symptoms in current smokers and in ex-smokers

**FIGURE 1:**
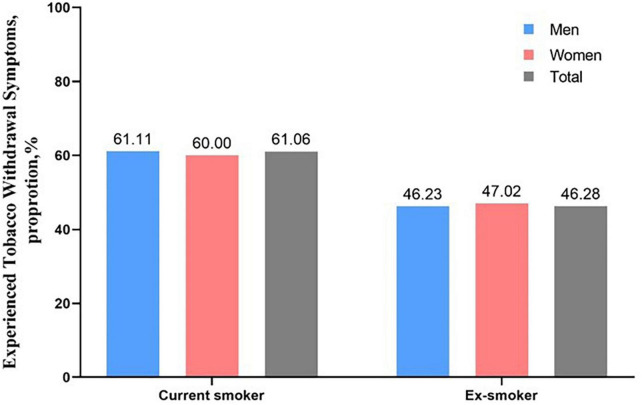
Experience of any withdrawal symptoms among male and female current smokers and ex-smokers.

Among current smokers, 11,993 (60.1%) experienced at least one withdrawal symptom, while among ex-smokers, this applied to 1,144 (46.3%, unadjusted OR = 1.76, 95% CI 1.62–1.93, *p* < 0.001). Adjusting for all baseline variables did not change the result (OR = 1.77, 95% CI 1.62–1.93, *p* < 0.001) Proportions of current smokers and ex-smokers who experienced any withdrawal symptoms were similar in men and women (see Figure 1).

Having experienced any withdrawal symptoms (present/absent) was correlated with FTND (*U* = 36.05, *p* < 0.001). There was also a correlation between FTND score and the number of withdrawal symptoms reported (Welch *F* = 452.93, *p* < 0.001).

Among current smokers, the most commonly-reported withdrawal symptoms were urge-to-smoke (34.01%), restlessness (26.82%), anxiety (21.03%), and difficulty concentrating (19.18%). The same four symptoms were most frequent in ex-smokers as well (see Table 2). Current smokers were more likely to report experiencing most of the listed withdrawal symptoms while ex-smokers were more likely to experience increased appetite and weight gain (11.93% vs. 6.37, *P* < 0.001).

**TABLE 2:** Withdrawal symptoms in smokers and ex-smokers.

Withdrawal symptoms	Current smokers (*n* = 19,643)	Ex-smoker (*n* = 2,472)	*p*
Urge-to-smoke	6,680 (34.01%)	616 (24.92%)	<0.001
Restlessness	5,269 (26.82%)	435 (17.60%)	<0.001
Anxiety	4,131 (21.03%)	381 (15.41%)	<0.001
Difficulty concentrating	3,768 (19.18%)	303 (12.26%)	<0.001
Irritability/Frustration/Anger	2,459 (12.52%)	251 (10.15%)	<0.001
Depression	2,672 (13.60%)	217 (8.78%)	<0.001
Sleep disturbance	1,117 (5.69%)	100 (4.05%)	<0.001
Tiredness	1,795 (9.14%)	156 (6.31%)	0.001
Increased appetite/Weight gain	1,252 (6.37%)	295 (11.93%)	<0.001
Mouth ulcers	276 (1.41%)	47 (1.90%)	0.053
Constipation	172 (0.88)	32 (1.29)	0.040
Others	1,225 (6.24%)	172 (6.96%)	0.172

Data are shown as number (%).

We also looked separately at the withdrawal experience in current smokers who tried to quit in the past and those that did not. Among 12,048 current smokers who attempted to quit in the past, 8,387 (69.9%) experienced at least one withdrawal symptom when unable to smoke. Among 7,595 who reported no previous quit attempts, 3,607 (47.5%) reported such experience. Supplementary Table 2 compares the experience of withdrawal symptoms in current smokers who did and did not attempt to stop smoking in the past. Urge-to-smoke and restlessness were the most common symptoms in both groups, but all individual withdrawal symptoms were more frequent in smokers with past unsuccessful quit attempts.

We assessed internal consistency of the Tobacco withdrawal symptoms checklist. The Cronbach’s alpha coefficient was 0.687, indicating an acceptable level of reliability (Supplementary Tables 5, 6). For the Chinese version of the Fagerström Test of Tobacco Dependence, the coefficient was 0.728.

### Predictors of withdrawal symptoms

Table 3 shows variables that were associated with whether current smokers and ex-smokers reported experiencing any withdrawal symptoms. Younger age, higher tobacco dependence and failed quit attempts were associated with withdrawal symptoms in both groups. In addition, poorer health and a higher number of pack years were associated with tobacco withdrawal in current smokers.

**TABLE 3:** Predictors of experiencing any withdrawal symptoms in current smokers and ex-smokers.

Variables	Current smokers				Ex-smokers			
	Unadjusted OR (95% CI)	*P*	Adjusted OR^†^ (95% CI)	*p*	Unadjusted OR (95% CI)	*P*	Adjusted OR* (95% CI)	*p*
Age		0.0190		<0.0001		0.3175		0.0205
Age, 10 years	1.05 (1.01–1.10)		0.86 (0.82–0.91)		0.94 (0.84–1.06)		0.84 (0.72–0.97)	
Sex		0.0853		0.6009		0.2483		0.3524
Women	1.00 (ref)		1.00 (ref)		1.00 (ref)		1.00 (ref)	
Men	1.21 (0.97–1.49)		1.06 (0.84–1.35)		1.33 (0.82–2.15)		1.31 (0.74–2.34)	
Residence		0.7946				0.1895		
Urban	1.00 (ref)				1.00 (ref)			
Rural	0.99 (0.89–1.09)				1.19 (0.92–1.55)			
Education level		0.5243				0.1939		
College or higher	1.00 (ref)				1.00 (ref)			
Middle or high school	0.99 (0.84–1.17)				0.78 (0.52–1.17)			
Primary school or less	1.05 (0.89–1.25)				0.99 (0.65–1.50)			
Marital status		0.0149		0.0237		0.6705		
Married	1.00 (ref)		1.00 (ref)		1.00 (ref)			
Single	0.75 (0.62–0.92)		0.78 (0.63–0.96)		0.78 (0.37–1.64)			
Separated/Divorced/Widowed	1.05 (0.88–1.24)		1.01 (0.84–1.20)		1.14 (0.72–1.82)			
Annual household income (RMB)		0.3027				0.6125		
≥50,000	1.00 (ref)				1.00 (ref)			
20,000–49,999	0.98 (0.87–1.11)				1.05 (0.76–1.44)			
<20,000	1.07 (0.95–1.21)				1.18 (0.85–1.64)			
Self-reported overall health status		<0.0001		<0.0001		0.0196		0.0764
Average	1.00 (ref)		1.00 (ref)		1.00 (ref)		1.00 (ref)	
Good	0.71 (0.64–0.79)		0.79 (0.70–0.89)		0.74 (0.56–0.99)		0.75 (0.53–1.05)	
Poor	1.17 (0.97–1.43)		1.07 (0.88–1.32)		1.27 (0.82–1.98)		1.37 (0.89–2.10)	
Smoking pack-years		<0.0001		<0.0001		0.0021		0.5338
Each 5 pack-years	1.11 (1.10–1.13)		1.07 (1.05–1.10)		1.05 (1.02–1.08		1.01 (0.97–1.05)	
Age at starting smoking		<0.0001		0.0722		0.0153		0.3441
Each 10 years	0.72 (0.66–0.78)		0.91 (0.83–1.00)		0.69 (0.50–0.93)		0.85 (0.61–1.19)	
FTND		<0.0001		<0.0001		<0.0001		<0.0001
0–3	1.00 (ref)		1.63 (1.45–1.83)		1.00 (ref)		1.00 (ref)	
4–6	2.07 (1.86–2.29)		2.47 (1.92–3.18)		1.92 (1.45–2.54)		1.78 (1.33–2.39)	
≥7	4.35 (3.52–5.38)				2.56 (1.59–4.11)		2.22 (1.26–3.91)	
Failed attempts to quit or cut down		<0.0001		<0.0001		<0.0001		<0.0001
No	1.00 (ref)		1.00 (ref)		1.00 (ref)		1.00 (ref)	
Yes	2.76 (2.48–3.06)		2.45 (2.20–2.73)		3.34 (2.54–4.41)		3.17 (2.39–4.21)	

Adjusted OR^†^: including age, sex, residence, education level, annual household income, self-reported overall health status, smoking pack-years, age at starting smoking, FTND, trying to quit or cut down but failing. Adjusted OR*: including age, sex, self-reported overall health status, smoking pack-years, age at starting smoking, FTND, failed attempts to quit or cut down.

### Predictors associated successful smoking cessation (being ex-smokers)

In another multivariable-adjusted analyses (Supplementary Table 3), successful smoking cessation (defined as being ex-smokers) was significantly associated with not experiencing withdrawal symptoms when they quit smoking (OR: 2.05, 95% CI: 1.86–2.27), along with lower smoking pack-years, higher education level, poor health status (*P* < 0.01).

## Discussion

To the best of our knowledge, this is the first and largest study to report the patterns of tobacco withdrawal experience among a representative sample of smokers and ex-smokers in China, and our findings fill several knowledge gaps about the proportion of smokers outside clinical settings whose smoking may be driven in part at least by the discomfort they experience when they try to limit or stop smoking. Approximately 60% of current smokers reported experiencing withdrawal discomfort when unable to smoke, while only 46% of ex-smokers reported such experience. The most frequent withdrawal symptoms in both current smokers and ex-smokers were urge to smoke, restlessness, anxiety, and irritability. Higher tobacco dependence and older age were associated with experiencing tobacco withdrawal in both groups, and poorer health and longer history of smoking were associated with tobacco withdrawal in current smokers.

Several studies reported tobacco withdrawal experience in different populations. In a US cohort of 554 adult smokers, 87% reported withdrawal symptoms after not smoking for 1 week and two-thirds reported craving (22). In a cohort of 2,862 male smokers in Japan, 67% reported ever experiencing tobacco withdrawal symptoms (23). In a cohort of 1,111 US adolescent smokers, over 50% experienced tobacco withdrawal symptoms, and a higher proportion of current smokers than successful quitters reported withdrawal symptoms in their most recent quit attempt (9). In our study, almost two-thirds of current smokers and one-third of ex-smokers experienced tobacco withdrawal symptoms. Although these studies are not entirely comparable, the findings indicate that the ubiquity of withdrawal discomfort is likely to contribute to the tenacity of smoking.

It is interesting to note that among Chinese ever-smokers, only 11% are ex-smokers, while among ever-smokers in Western countries such as UK and USA, over 60% are ex-smokers (24, 25). Ex-smokers can be expected to be recruited primarily from smokers who were less dependent and less likely to experience withdrawal discomfort. This was indeed the case in our Chinese sample, where ex-smokers showed fewer signs of dependence than current smokers. Similarly, among current smokers, those motivated to quit but still smoking were more likely to report experiencing withdrawal symptoms when unable to smoke than those who did not try to quit. If the same dynamics applies in Western countries, it is likely that among the much lower proportion of the local adult population who continue to smoke despite the widespread awareness of the risks of smoking and the fact that the majority of smokers have already quit, an even higher proportion can be expected to suffer from withdrawal discomfort when unable to smoke. The estimate from the Chinese data that this affects 60% of all current smokers and 70% of those with past quit attempts may well be conservative.

Among individual withdrawal symptoms, the high occurrence of anxiety was unexpected. A 2004 review (26) noted that an increase in anxiety was reported in some studies but not in others and considered anxiety an uncertain tobacco withdrawal symptom, requiring further research. The review, however, also noted the cross-cultural differences in the concept of anxiety and different meanings that can be attached to particular words in different languages. It is possible that the semantic meaning of the Chinese word for anxiety corresponds more closely with withdrawal experience than the English expression.

It should be noted that our data come from China, a country with a high prevalence of smoking in men, very low prevalence of smoking in women, and a more limited tobacco control history than in Western countries. In the current sample, 95% of smokers were men. This corresponds with other sources of data on smoking prevalence in China from the same time period that report that 49% of men and 3% of women were smoking (27). The incidence of withdrawal experience was the same across the sexes and so the predominance of men is unlikely to have a substantial effect on the generalizability of the results.

Several limitations of our study should be considered while interpreting the results. The findings are based on self-reports that may suffer from various recall biases. The survey assessed whether ever-smokers experienced any tobacco withdrawal symptoms when unable to smoke; and if so, which symptoms they experienced. However, there was no assessment of symptoms’ severity. Future studies should consider including this qualitative aspect. A recall of the withdrawal experience may have differed in current smokers and ex-smokers. At least some ex-smokers would be recalling events from a more distant past; and the recall could have been colored by a perception of success and failure in quitting smoking. The fact that in comparison to Western countries, smoking in China is much more common and much less stigmatized may ameliorate this concern, but it may still have played a role. The question about withdrawal symptoms did not specify the duration of abstinence or situations in which withdrawal symptoms were experienced. Future studies may consider asking specifically about symptoms experienced during short-term and long-term periods of abstinence from smoking and about specific situations in which the symptoms were noticed. The study design was cross-sectional and no causal interpretations can be made. We also did not assess the effects of alcohol and other substance use, past use of smoking cessation treatments, or indicators of mental health, particularly levels of anxiety. Future studies should consider examining the impact of these variables.

## Conclusion

Withdrawal discomfort represents one of the main obstacles to smoking cessation and it is one of the key targets of stop-smoking treatments (28). Experiencing tobacco withdrawal symptoms is likely to negatively impact smokers’ ability to stop tobacco use. Our study documents for the first time that such experience is common in adult smokers in the general population in China. The ubiquity of withdrawal discomfort is likely to contribute to the tenacity of smoking. It also suggests that interventions that reduce withdrawal discomfort and that can be disseminated on population scale may help to reduce smoking.

## Data availability statement

The raw data supporting the conclusions of this article will be made available by the authors, without undue reservation.

## Ethics statement

The studies involving human participants were reviewed and approved by the Ethics Review Committees of the China Health Education Center (Beijing, China). The patients/participants provided their written informed consent to participate in this study.

## Author contributions

CW, Y-HL, and DX conceived and designed the study. DX supervised the study. DX, Z-MC, PH, Z-YC, and ZL drafted the report. X-MZ, X-QN, LL, and FP conducted the statistical analysis. All authors contributed to the interpretation of the findings and manuscript preparation, revised the report, and approved the final version before submission.
