# Supplementary material for: The experience of tobacco withdrawal symptoms among current smokers and ex-smokers in the general population: Findings from nationwide China Health Literacy Survey during 2018-19
Source: Front Psychiatry. 2023 Jan 13;13:1023756. doi: 10.3389/fpsyt.2022.1023756 (PMC9880314; doi:10.3389/fpsyt.2022.1023756)
Supplement: Supplementary file 1 [file Data_Sheet_1.docx]

Supplementary Material


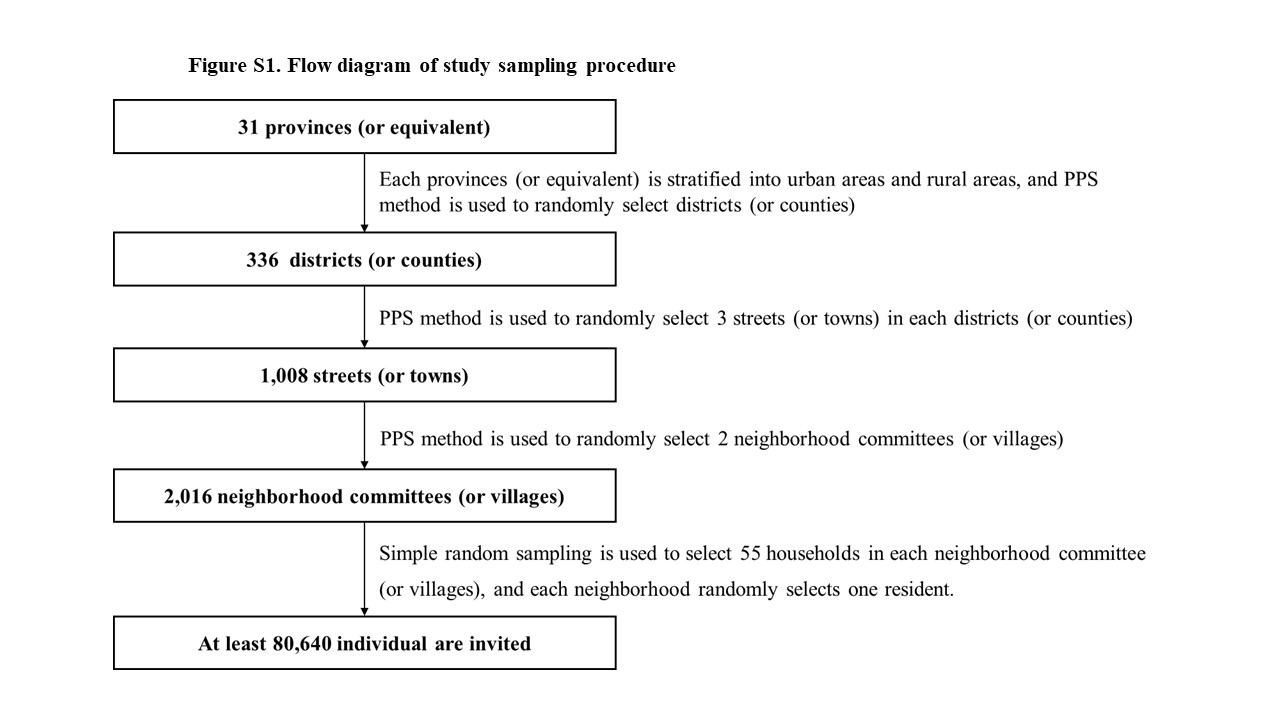


**Supplementary Figure 1 (Figure S1). Flow diagram of study sampling procedure**


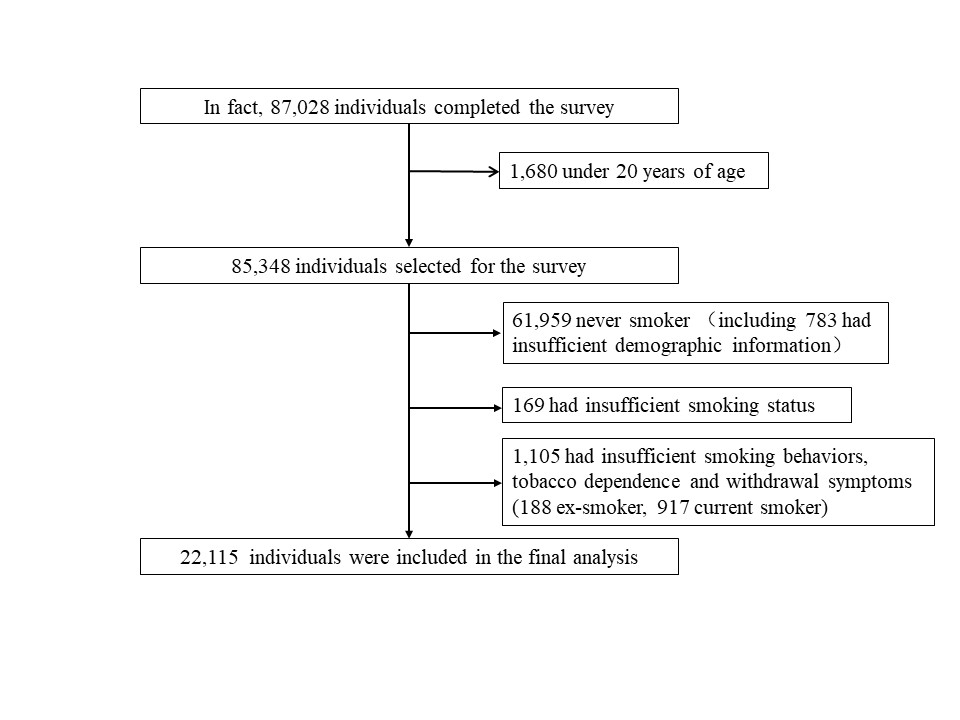


**Supplementary Figure 2(Figure S2). Flow diagram of participants through the study**

**Table S1. Smoking characteristics of the study population**

| **Variables** | **Current smokers（n=19,643）** | **Ex-smokers（n=2,472）** | p |
| --- | --- | --- | --- |
| **Age started smoking** |  |  |  |
| Median (IQR) | 20.00 (6.00) | 20.00 (5.00) | 0.084 |
| **Years of smoking** |  |  |  |
| Median (IQR) | 28.92 (19.33) | 23.33 (21.58) | <0.001 |
| **Cigarettes per day** |  |  |  |
| Median (IQR) | 15.00 (10.00) | 13.00 (13.00) | <0.001 |
| **Feels dependent on tobacco N (%)** |  |  | <0.001 |
| Yes | 14,203 (72.31) | 1,112 (44.98) |  |
| No | 5,440 (27.69) | 1,360 (55.02) |  |
| **Smoking more than intended to N (%)** |  |  | <0.001 |
| Yes | 6,113 (31.12) | 432 (17.48) |  |
| No | 9,921 (50.51) | 1,513 (61.21) |  |
| Not sure | 3,609 (18.37) | 527 (21.32) |  |
| **Smoking after it caused physical health problems N (%)** |  |  | <0.001 |
| Yes | 5,755 (29.30) | 392 (15.86) |  |
| No | 13,888 (70.70) | 2,080 (84.14) |  |
| **Smoking after it caused mental health problems N (%)** |  |  | <0.001 |
| Yes | 5,519 (28.10) | 397 (16.06) |  |
| No | 14,124 (71.90) | 2,075 (83.94) |  |
| **Tried to quit or cut down on tobacco but failed N (%)** |  |  | <0.001 |
| Yes | 12,048 (61.33) | 1,076 (43.53) |  |
| No | 7,595 (38.67) | 1,396 (56.47) |  |
| **FTND (%)** |  |  | <0.001 |
| 0-3 | 10,048 (51.15) | 1,510 (61.08) |  |
| 4-6 | 8,180 (41.64) | 818 (33.09) |  |
| ≥7 | 1,415 (7.20) | 144 (5.83) |  |
| Median (IQR) | 3.46 (1.96) | 2.94 (1.95) | <0.001 |

Note: Data are shown as number (%), median (IQR) or mean (SE).

**Table S2. Withdrawal symptoms in current smokers who did and did not report past quit attempts**

| **Withdrawal symptom** | **Current smoker** | | **p** |
| --- | --- | --- | --- |
|  | Tried to quit N=12,048(61.33%) | Did not try to quit N**=**7,595(38.67%) |  |
| Urge-to-smoke | 4,619 (38.3%) | 2,061 (27.1%) | <0.001 |
| Restlessness | 3,884 (32.2%) | 1,385 (18.2%) | <0.001 |
| Anxiety | 3,055 (25.4%) | 1,076 (12.4%) | <0.001 |
| Difficulty concentrating | 2,739 (22.7%) | 1,029 (13.5%) | <0.001 |
| Irritability/frustration/anger | 1,837 (15.2%) | 622 (8.2%) | <0.001 |
| Depression | 1,971 (16.4%) | 701 (9.2%) | <0.001 |
| Sleep disturbance | 1,302 (10.8%) | 493 (6.5%) | <0.001 |
| Tiredness | 817 (6.8%) | 300 (3.9%) | <0.001 |
| Increased appetite/ weight gain | 988 (8.2%) | 264 (3.5%) | <0.001 |
| Mouth ulcers | 213 (1.8%) | 63 (0.8%) | <0.001 |
| Constipation | 123 (1.0%) | 49 (0.6%) | 0.006 |
| Others | 860 (7.1%) | 365 (4.8%) | <0.001 |

Note: Data are shown as number (%).

**Table S3. Adjusted ORs for ex-smokers in ever smokers aged 20-69years**

| Variables | Man | | Women | | Total | |
| --- | --- | --- | --- | --- | --- | --- |
|  | OR  (95% CI) | P | OR  (95% CI) | P | OR  (95% CI) | P |
| **Age group** |  |  |  |  |  |  |
| 20-29 | 1.00 (Ref) |  | 1.00 (Ref) |  | 1.00 (Ref) |  |
| 30-39 | 1.21  (0.90-1.65) | 0.2216 | 1.03  (0.37-3.06) | 0.9509 | 1.18  (0.88-1.58) | 0.2703 |
| 40-49 | 1.19  (0.89-1.60) | 0.2546 | 0.67  (0.24-2.06) | 0.4715 | 1.13  (0.86-1.50) | 0.3897 |
| 50-59 | 1.55  (1.17-2.08) | 0.0031 | 0.31  (0.11-0.95) | 0.0332 | 1.41  (1.08-1.87) | 0.0142 |
| 60-69 | 2.85  (2.15-3.84) | <0.0001 | 0.54  (0.19-1.63) | 0.2524 | 2.58  (1.97-3.42) | <0.0001 |
| **Gender** |  |  |  |  |  |  |
| Women | 1.00 (Ref) |  | 1.00 (Ref) |  | 1.00 (Ref) |  |
| Men |  |  |  |  | 0.74  (0.60-0.93) | 0.0073 |
| **Urbanization** |  |  |  |  |  |  |
| Rural | 1.00 (Ref) |  | 1.00 (Ref) |  | 1.00 (Ref) |  |
| Urban | 0.94  (0.85-1.05) | 0.2742 | 1.37  (0.84-2.30) | 0.2176 | 0.95  (0.86-1.06) | 0.3406 |
| **Education level** |  |  |  |  |  |  |
| Primary school or less | 1.00 (Ref) |  | 1.00 (Ref) |  | 1.00 (Ref) |  |
| Middle or high school | 1.28  (1.14-1.45) | <0.0001 | 1.13  (0.66-1.92) | 0.6492 | 1.29  (1.15-1.45) | <0.0001 |
| College or higher | 1.67  (1.37-2.04) | <0.0001 | 1.67  (0.62-4.34) | 0.2982 | 1.69  (1.38-2.05) | <0.0001 |
| **Annual family income** |  |  |  |  |  |  |
| <20000 | 1.00 (Ref) |  | 1.00 (Ref) |  | 1.00 (Ref) |  |
| 20000-49999 | 0.80  (0.70-0.91) | <0.0001 | 0.92  (0.49-1.75) | 0.8049 | 0.80  (0.71-0.90) | 0.0004 |
| ≥50000 | 0.72  (0.62-0.82) | <0.0001 | 0.79  (0.42-1.50) | 0.4668 | 0.72  (0.63-0.82) | <0.0001 |
| **Self-reported health status** |  |  |  |  |  |  |
| Average | 1.00 (Ref) |  | 1.00 (Ref) |  | 1.00 (Ref) |  |
| Poor | 1.58  (1.33-1.89) | <0.0001 | 1.45  (0.77-2.65) | 0.2392 | 1.56  (1.32-1.85) | <0.0001 |
| Good | 0.78  (0.70-0.87) | <0.0001 | 0.76  (0.47-1.24) | 0.2721 | 0.78  (0.70-0.87) | <0.0001 |
| **Smoking pack-years** |  |  |  |  |  |  |
| ≥20 | 1.00 (Ref) |  | 1.00 (Ref) |  | 1.00 (Ref) |  |
| 10-19 | 1.53  (1.33-1.75) | <.0001 | 1.33  (0.67-2.60) | 0.4045 | 1.50  (1.31-1.71) | <.0001 |
| <10 | 3.64  (3.18-4.16) | <.0001 | 3.19  (1.83-5.71) | <.0001 | 3.57  (3.14-4.07) | <.0001 |
| **Cigarettes smoked per day** |  |  |  |  |  |  |
| ≥20 | 1.00 (Ref) |  | 1.00 (Ref) |  | 1.00 (Ref) |  |
| 10-19 | 1.09  (0.96-1.23) | 0.1739 | 1.62  (0.88-3.03) | 0.1219 | 1.10  (0.98-1.24) | 0.1170 |
| <10 | 2.11  (1.85-2.41) | <.0001 | 3.09  (1.77-5.57) | 0.0001 | 2.15  (1.89-2.44) | <.0001 |
| **Smoking starting age** |  |  |  |  |  |  |
| ≥25 | 1.00 (Ref) |  | 1.00 (Ref) |  | 1.00 (Ref) |  |
| 20-24 | 1.19  (1.04-1.36) | 0.0119 | 1.94  (1.15-3.28) | 0.0131 | 1.23  (1.08-1.40) | <.0001 |
| <20 | 1.06  (0.93-1.21) | 0.3716 | 1.21  (0.69-2.12) | 0.5019 | 1.08  (0.95-1.22) | <.0001 |
| **Smoking duration** |  |  |  |  |  |  |
| ≥20 | 1.00 (Ref) |  | 1.00 (Ref) |  | 1.00 (Ref) |  |
| 10-19 | 3.58  (3.06-4.18) | <.0001 | 1.45  (0.79-2.59) | 0.2213 | 3.33  (2.87-3.87) | <.0001 |
| 1-9 | 11.34  (9.10-14.13) | <.0001 | 3.29  (1.55-6.86) | 0.0017 | 10.32  (8.38-12.73) | <.0001 |
| **Withdrawal Symptoms** |  |  |  |  |  |  |
| Yes | 1.00 (Ref) |  | 1.00 (Ref) |  | 1.00 (Ref) |  |
| No | 2.08  (1.88-2.30) | <0.001 | 1.62  (1.04-2.50) | 0.031 | 2.05  (1.86-2.27) | <0.001 |

Note: adjusted including the co-variables sex, age, gender, age, urbanization, education level, ethnicity, marriage status, annual family yearly income, self-reported overall health status.

**Table S4. Predictors of experiencing any withdrawal symptoms in current smokers and ex-smokers**

| Variables | Current smokers | | | |  | Ex-smokers | | | |
| --- | --- | --- | --- | --- | --- | --- | --- | --- | --- |
|  | Unadjusted OR  (95% CI) | p | Adjusted OR†  (95% CI) | p |  | Unadjusted OR  (95% CI) | p | Adjusted OR*  (95% CI) | p |
| **Age** |  | 0.3972 |  | <0.0001 |  |  | 0.0017 |  | <0.0001 |
| Age, 10 years | 1.01  (0.99-1.04) |  | 0.89  (0.86-0.92) |  |  | 0.89  (0.84-0.96) |  | 0.83  (0.77-0.91) |  |
| **Sex** |  | 0.0418 |  | 0.9238 |  |  | 0.6253 |  | 0.4597 |
| Women | 1.00 (ref) |  | 1.00 (ref) |  |  | 1.00 (ref) |  | 1.00 (ref) |  |
| Men | 1.14  (1.01-1.30) |  | 1.01  (0.88-1.16) |  |  | 0.92  (0.66-1.28) |  | 0.87  (0.61-1.25) |  |
| **Residence** |  | <0.0001 |  | 0.0009 |  |  | 0.2816 |  |  |
| Urban | 1.00 (ref) |  | 1.00 (ref) |  |  | 1.00 (ref) |  |  |  |
| Rural | 1.15  (1.09-1.22) |  | 1.11  (1.05-1.19) |  |  | 1.09  (0.93-1.28) |  |  |  |
| **Education level** |  | 0.0206 |  | <0.0001 |  |  | 0.6425 |  |  |
| College or higher | 1.00 (ref) |  | 1.00 (ref) |  |  | 1.00 (ref) |  |  |  |
| Middle or high school | 0.97  (0.88-1.06) |  | 0.78  (0.70-0.87) |  |  | 0.91  (0.71-1.17) |  |  |  |
| Primary school or less | 1.05  (0.95-1.17) |  | 0.79  (0.70-0.89) |  |  | 0.98  (0.75-1.27) |  |  |  |
| **Marital status** |  | 0.4453 |  |  |  |  | 0.6978 |  |  |
| Married | 1.00 (ref) |  |  |  |  | 1.00 (ref) |  |  |  |
| Single | 0.94  (0.84-1.05) |  |  |  |  | 0.85  (0.57-1.25) |  |  |  |
| Separated/divorced/widowed | 1.02  (0.92-1.14) |  |  |  |  | 1.00  (0.74-1.35) |  |  |  |
| **Annual household income (RMB)** |  | <0.0001 |  | 0.0123 |  |  | 0.2589 |  |  |
| ≥50000 | 1.00 (ref) |  | 1.00 (ref) |  |  | 1.00 (ref) |  |  |  |
| 20000-49999 | 1.10  (1.03-1.18) |  | 1.06  (0.99-1.15) |  |  | 1.03  (0.85-1.25) |  |  |  |
| <20000 | 1.19  (1.11-1.28) |  | 1.13  (1.04-1.23) |  |  | 1.17  (0.97-1.42) |  |  |  |
| **Self-reported overall health status** |  | <0.0001 |  | <0.0001 |  |  | 0.0132 |  | 0.0194 |
| Average | 1.00 (ref) |  | 1.00 (ref) |  |  | 1.00 (ref) |  | 1.00 (ref) |  |
| Good | 0.74  (0.70-0.79) |  | 0.78  (0.73-0.83) |  |  | 0.86  (0.07-0.90) |  | 0.88  (0.73-1.05) |  |
| Poor | 1.15  (0.97-1.43) |  | 1.04  (0.91-1.19) |  |  | 1.24  (0.95-1.61) |  | 1.29  (0.97-1.72) |  |
| **Smoking pack-years** |  | <0.0001 |  | <0.0001 |  |  | <0.0001 |  | 0.2947 |
| Each 5 pack-years | 1.09  (1.09-1.10) |  | 1.05  (1.04-1.07) |  |  | 1.05  (1.03-1.07) |  | 1.01  (0.99-1.04) |  |
| **Age at starting smoking** |  | <0.0001 |  | <0.0001 |  |  | <0.0001 |  | 0.0013 |
| Each 10 years | 0.72  (0.68-0.75) |  | 0.88  (0.83-0.93) |  |  | 0.63  (0.54-0.74) |  | 0.76  (0.64-0.90) |  |
| **FTND** |  | <0.0001 |  | <0.0001 |  |  | <0.0001 |  | <0.0001 |
| 0-3 | 1.00 (ref) |  | 1.00 (ref) |  |  | 1.00 (ref) |  | 1.00 (ref) |  |
| 4-6 | 2.05  (1.93-2.18) |  | 1.67  (1.56-1.79) |  |  | 2.10  (1.77-2.50) |  | 1.83  (1.51-2.22) |  |
| ≥7 | 3.48  (1.93-2.18) |  | 2.18  (1.85-2.57) |  |  | 2.97  (2.09-4.29) |  | 2.55  (1.65-3.97) |  |
| **Failed attempts to quit or cut down** |  | <0.0001 |  | <0.0001 |  |  | <0.0001 |  | <0.0001 |
| No | 1.00 (ref) |  | 1.00 (ref) |  |  | 1.00 (ref) |  | 1.00 (ref) |  |
| Yes | 2.53  (2.39-2.69) |  | 2.33  (2.19-2.48) |  |  | 3.53  (2.99-4.17) |  | 3.27  (2.76-3.88) |  |

Note: Adjusted OR†: including age, sex, residence, education level, annual household income, self-reported overall health status, smoking pack-years, age at starting smoking, FTND, trying to quit or cut down but failing. Adjusted OR*: including age, sex, self-reported overall health status, smoking pack-years, age at starting smoking, FTND, failed attempts to quit or cut down.

**Table S5. The Zero-order correlation of FTND subscales in ever smokers.**

| **Subscales** | **1** | **2** | **3** | **4** | **5** | **6** |
| --- | --- | --- | --- | --- | --- | --- |
| 1. How soon after you wake up do you smoke your first cigarette | 1 |  |  |  |  |  |
| 2. Do you find it difficult to refrain from smoking in places where it is forbidden | 0.36 | 1 |  |  |  |  |
| 3. Which cigarette would you hate most to give up? | 0.56 | 0.35 | 1 |  |  |  |
| 4. How many cigarettes/day do you smoke? | 0.51 | 0.42 | 0.26 | 1 |  |  |
| 5. Do you smoke more frequently during the first hours after waking than during the rest of day? | 0.40 | 0.24 | 0.37 | 0.33 | 1 |  |
| 6. Do you smoking if you are so ill that you are in bed most of the day? | 0.38 | 0.29 | 0.15 | 0.32 | 0.50 | 1 |

**Table S6. The Zero-order correlation of Tobacco Withdrawal Symptoms subscales in ever smokers.**

| **Subscales** | **1** | **2** | **3** | **4** | **5** | **6** | **7** | **8** | **9** | **10** | **11** | **12** |
| --- | --- | --- | --- | --- | --- | --- | --- | --- | --- | --- | --- | --- |
| 1. Urge-to-smoke | 1 |  |  |  |  |  |  |  |  |  |  |  |
| 2. Restlessness | 0.28 | 1 |  |  |  |  |  |  |  |  |  |  |
| 3. Anxiety | 0.23 | 0.23 | 1 |  |  |  |  |  |  |  |  |  |
| 4. Difficulty concentrating | 0.25 | 0.23 | 0.25 | 1 |  |  |  |  |  |  |  |  |
| 5. Irritability/frustration/anger | 0.12 | 0.19 | 0.17 | 0.16 | 1 |  |  |  |  |  |  |  |
| 6. Depression | 0.09 | 0.16 | 0.26 | 0.20 | 0.17 | 1 |  |  |  |  |  |  |
| 7. Sleep disturbance | 0.08 | 0.13 | 0.12 | 0.11 | 0.13 | 0.15 | 1 |  |  |  |  |  |
| 8. Tiredness | 0.10 | 0.07 | 0.70 | 0.11 | 0.07 | 0.08 | 0.11 | 1 |  |  |  |  |
| 9. Increased appetite/ weight gain | 0.06 | 0.04 | 0.04 | 0.06 | 0.05 | 0.05 | 0.08 | 0.09 | 1 |  |  |  |
| 10. Mouth ulcers | 0.01 | 0.04 | 0.06 | 0.05 | 0.07 | 0.06 | 0.08 | 0.07 | 0.06 | 1 |  |  |
| 11. Constipation | 0.05 | 0.03 | 0.53 | 0.06 | 0.06 | 0.06 | 0.07 | 0.08 | 0.10 | 0.19 | 1 |  |
| 12. Others | 0.05 | 0.06 | 0.08 | 0.07 | 0.10 | 0.09 | 0.16 | 0.09 | 0.09 | 0.13 | 0.20 | 1 |
